# Etiological factors of the *risk for occupational illness* in nursing professionals: An etiology and risk review protocol

**DOI:** 10.1371/journal.pone.0335976

**Published:** 2026-01-07

**Authors:** Cyntia Leenara Bezerra da Silva Gonzaga, Ana Clara Dantas, Bárbara Ebilizarda Coutinho Borges, Leandro Melo de Carvalho, Heloise Rosália de Carvalho Nascimento, Maria Clara Regis de Souza, Belo Mario Luis Generoso, Allyne Fortes Vitor

**Affiliations:** Department of Nursing, Federal University of Rio Grande do Norte, Natal, Rio Grande do Norte, Brazil; Federal University of Rio Grande do Sul: Universidade Federal do Rio Grande do Sul, BRAZIL

## Abstract

**Introduction:**

Nursing professionals are inherently exposed to various risk factors, which may be exacerbated depending on the conditions under which care is provided. For instance, many healthcare institutions are considered hazardous environments for admitting patients with diverse diseases and performing procedures that carry potential risks of accidents and occupational diseases with them.

**Objective:**

To identify the etiological factors associated with the *Risk for Occupational Illness* Nursing diagnosis in Nursing professionals.

**Methods:**

An Etiology and Risk Review Protocol was developed following the guidelines outlined in the JBI Manual for Evidence Synthesis and registered in the International Prospective Register of Systematic Reviews under number CRD42024549181.

**Results:**

The findings of this review will be publicly disseminated through scientific journals in health sciences, contributing to enhancing the evidence base corresponding to the *Risk for Occupational Illness* Nursing diagnosis. This will support its inclusion in future NANDA International editions, ensuring that the diagnosis remains current and aligned with the latest scientific literature.

**Conclusion:**

It is anticipated that the review will generate evidence addressing stages 3, 4, 7, and 8 of the concept analysis for the *Risk for Occupational Illness* diagnosis, specifically: Identifying possible uses of the concept; Determining critical defining attributes; Identifying antecedents and consequences; and Defining empirical referents.

**Descriptors:**

Occupational stress; Occupational diseases; Etiology; Nursing diagnosis; Nursing professionals

## Introduction

Workers’ health encompasses the interactions between work and the health-disease process and is regulated by the Organic Health Law (Law No. 8080/90), which establishes that the State must ensure favorable conditions for the full performance of work activities. Accordingly, economic and social policies aimed at reducing the risk of illness and other health-related harms to workers are essential. In this context, the regulation defines the conditions for healthcare provision through health surveillance, as well as for workers’ health promotion, protection and recovery [1].

Nursing professionals provide continuous healthcare services 24 hours a day, oftentimes under conditions marked by limited resources and precarious labor structures. They typically work between 30 and 40 hours per week and have more than one employment contract in many cases [2]. Combined with shift work, these factors contribute to physical and emotional exhaustion and interpersonal conflicts in the workplace, which negatively affect both physical and psychological well-being in these workers. As a result, occupational illnesses—many of which go unnoticed or are underestimated by the professionals themselves—are intensified.

Globally, work-related accidents and occupational diseases represent a substantial burden on Public Health, accounting for up to 4% of the global GDP and resulting in 2.9 million annual deaths in 2019, a 26% increase since 2014 [3–4]. Healthcare professionals, particularly nurses, are disproportionately affected due to direct exposure to biological, chemical, ergonomic and psychosocial hazards. Work-related musculoskeletal disorders are highly prevalent among nurses, with 77.2% annual prevalence and lower back pain as the most common condition, with rates ranging from 32.5% to 87.5% [5–6].

A total of 329,176 work-related accidents involving exposure to biological materials were reported in Brazil between 2018 and 2022, with 54.4% affecting Nursing professionals [7]. Additionally, a growing number of occupational benefits were granted due to serious injuries, musculoskeletal disorders and mental health conditions [3,7].

The professional practice carries with it the potential to cause harms to workers, and occurrence of such harms is influenced by both the likelihood and severity of the risks involved. Recognizing that occupational exposure arises from the interaction between professionals and their work environment enables nurses to develop a prevention-focused mindset and implement strategies to reduce the risk for occupational illness [3]. Understanding the etiological and risk factors associated with occupational illness among Nursing professionals can inform the development of targeted preventive strategies, guide workplace safety policies and support evidence-based interventions within healthcare institutions.

In order to develop appropriate care plans and achieve the expected health outcomes, nurses rely on the Nursing Process (NP). The NP supports identifying, understanding, measuring and predicting individual health problems, thereby guiding the creation of effective and applicable care strategies. It consists of five interrelated, interdependent and cyclical stages: Nursing Assessment, Nursing Diagnosis, Nursing Planning, Nursing Implementation, and Nursing Evaluation [8–9].

In the NANDA International (NANDA-I) taxonomy, the *Risk for Occupational Illness* (00404) Nursing Diagnosis (ND) approved in 2023 belongs to Domain 11. Safety/Protection, and to Class 4. Environmental risks. This diagnosis includes 29 risk factors classified as individual factors, such as: (1) Difficulty with decision-making, (2) Excessive stress, (3) Improper use of Personal Protective Equipment, (4) Inaccurate follow-through of employee health protocol, (5) Inaccurate follow-through of safety protocol, (6) Inadequate action to address modifiable factors, (7) Inadequate communication skills, (8) Inadequate knowledge of modifiable factors, (9) Inadequate social support, (10) Inadequate understanding of the importance of Personal Protective Equipment, (11) Inadequate vaccination, (12) Inattentive to ergonomic principles, or (13) Ineffective weight management [10].

Additionally, there are environmental factors such as the following: (1) Conflicted labor relationships, (2) Excessive workload, (3) Exposure to chemical agents, (4) Exposure to biological agents, (5) Exposure to intermittent impacts, (6) Exposure to psychosocial agents, (7) Exposure to repetitive motion activities, (8) Inadequate access to Personal Protective Equipment, (9) Inadequate adoption of ergonomic principles, (10) Inadequate biological monitoring, (11) Inadequate dosimetry monitoring, (12) Inadequate employee health protocol, (13) Inadequate placement of collective protective equipment, (14) Inadequate safety protocol, (15) Ineffective workload management, or (16) Pathogen exposure [10].

The diagnosis also identifies the at-risk population: (1) Chestfeeding individuals, (2) Individuals whose work is comprised of monotonous activities, (3) Individuals with a history of physical trauma, (4) Individuals with a history of traumatic professional exposure, (5) Individuals with a history of work-related accidents, (6) Individuals with limited access to healthcare services, (7) Individuals with multiple employment contracts, (8) Individuals with responsibilities beyond their own work ability, (9) Individuals with work-life imbalance, (10) Pregnant individuals, or (11) Rotating shift workers [10].

This Nursing diagnosis is defined as susceptibility to a work-related condition or disorder resulting from a non-instantaneous event or exposure. It currently holds a level of evidence of 2.1, which corresponds to the initial level assigned to an approved diagnosis within the NANDA-I taxonomy [10].

Although NANDA-I is a widely recognized classification system that seeks to standardize Nursing language through diagnostic categories, it requires continuous theoretical, clinical and content validation studies. Such efforts are essential for updating the body of evidence, ensuring accuracy and validity of the diagnoses and promoting a broader application of the taxonomy across diverse healthcare settings and populations [11].

The process of validating Nursing diagnoses involves three sequential stages: Concept analysis, Content validation by experts and Clinical validation. Among them, reviewing the etiological and risk factors is fundamental for the concept analysis stage, which serves as the starting point for the validation process [12].

Based on this rationale, the following research question was formulated: Which are the etiological factors associated with the *Risk for Occupational Illness* Nursing diagnosis in Nursing professionals?

Therefore, the objective of this study was to identify the etiological factors associated with the *Risk for Occupational Illness* Nursing diagnosis among Nursing professionals.

## Method

This is a protocol for an Etiology and Risk Review, developed in accordance with the PRISMA-P (Preferred Reporting Items for Systematic Review and Meta-Analysis Protocols) guidelines and the methodological recommendations provided in Chapter 7 of the JBI Manual for Evidence Synthesis. The protocol has been registered in the International Prospective Register of Systematic Reviews (PROSPERO) under number CRD42024549181 [13] (S1 File).

The review will follow these stages: Formulation of the research question, Identification of relevant studies, Assessment of methodological quality and data extraction, and Synthesis of the results [14].

This systematic review aims at supporting the development of a concept analysis for the *Risk for Occupational Illness* Nursing diagnosis The review will be structured using the PEO mnemonic, namely: P (Population); E (Etiology); and O (Outcome) [13]. Accordingly, the elements considered are P (Nursing professionals), E (Etiological factors) and O (*Risk for Occupational Illness*). The guiding research question is as follows: “Which are the etiological factors associated with the “*Risk for Occupational Illness*” Nursing diagnosis in Nursing professionals?”

### Data sources

The literature search will be conducted through the CAPES Journals Portal, using the Federated Academic Community (*Comunidade Acadêmica Federada*, CAFe) platform belonging to the Federal University of Rio Grande do Norte (*Universidade Federal do Rio Grande do Norte*, UFRN). The following databases will be systematically searched: CINAHL (EBSCO), MEDLINE/PubMed (via the National Library of Medicine), Cochrane Library, Scopus (Elsevier), EMBASE and Latin American and Caribbean Literature in Health Sciences (*Literatura Latino-Americana em Ciências da Saúde*, LILACS). In addition, Gray Literature and official reports will be searched on the websites of the Spanish National Institutes for Occupational Health and Safety (*Instituto Nacional de Seguridad y Salud en el Trabajo*, INSST) and for Occupational Health (*Instituto Nacional de Seguridad en el Trabajo*, INST) to allow for a comparative analysis between the Brazilian and Spanish contexts, considering that the first Nursing diagnosis related to occupational health was developed by a Spanish scholar.

Importantly, no restrictions will be applied with regard to language or year of publication in the literature search.

### Search strategy

Selection of the search terms was guided by consultations in the Medical Subject Headings (MeSH) and the Descriptors in Health Sciences (*Descritores em Ciências da Saúde*, DeCS). A total of seven descriptors and one keyword were selected based on relevance to the research phenomenon, aiming to enhance comprehensiveness and sensitivity of the literature search (Table 1).

**Table 1:** MeSH/DeCS-indexed descriptors and keyword used in the search strategy.

PEO MNEMONIC STRATEGY	DeCS DESCRIPTORS	MESH DESCRIPTORS	KEYWORD
**P:** Nursing professionals	“Nursing professionals”	“Nursing professionals” “Nursing Team”	–
**E:** Etiological factors	“Etiology”	“Causality” “Etiology”	–
**O:** Risk for occupational illness	“Nursing diagnosis” “Occupational risks” “Occupational exposure” “Occupational stress” “Occupational diseases”	“Nursing diagnosis” “Occupational risks” “Occupational exposure” “Occupational stress” “Occupational diseases”	“Risk for occupational illness”

Boolean operators “AND” and “OR” were employed to combine the elements defined by the PEO framework [15]. The resulting search string constructed using these operators was as follows: “Nursing professionals” OR “Nursing team” AND “Causality” OR “Etiology” AND “Nursing diagnosis” OR “Occupational risks” OR “Occupational exposure” OR “Occupational stress” OR “Occupational diseases” OR “*Risk for occupational illness*”.

Given the unique indexing structures of each database and the objective of maximizing retrieval of relevant scientific evidence, the descriptors were strategically combined to generate tailored search strategies, herein referred to as syntax-product combinations (Table 2).

**Table 2:** Customized search syntaxes applied to each data source.

Data source	Search syntaxes
Scientific Electronic Library Online (SciELO)	(*“nursing professionals”) AND (“Occupational Exposure”) OR (“Occupational stress”) OR (“Occupational diseases”) OR (“Risk for occupational illness”)
Web of Science	TS=(“Nursing professionals”) OR TS=(“Nursing Team”) AND TS=(“Causality”) OR TS=(“Etiology”) AND TS=(“Nursing diagnosis”) OR TS=(“Occupational Exposure”) OR TS=(“Occupational stress”) OR TS=(“Occupational diseases”) OR TS=(“Risk for occupational illness”)
ScienceDirect	(“Nursing professionals”) AND (“Etiology”) AND ((“Nursing diagnosis”) OR (“Occupational Exposure”) OR (“Occupational stress”) OR (“Occupational diseases”) OR (“Risk for occupational illness”))
CINAHL (EBSCO)	TX(“Nursing professionals”) AND TX(“Etiology”) AND TX(“Occupational Risks”) OR TX(“Risk for occupational illness”)
MEDLINE/PubMed	((“Nursing professionals”) OR (“Nursing Team”)) AND ((“Causality”) OR (“Etiology”)) AND ((“Nursing diagnosis”) OR (“Occupational Risks”) OR (“Occupational Exposure”) OR (“Occupational stress”) OR (“Occupational diseases”) OR (“Risk for occupational illness”))
Scopus (Elsevier)	TITLE-ABS-KEY ((“Nursing professionals”) OR (“Nursing Team”)) AND ((“Causality”) OR (“Etiology”)) AND ((“Nursing diagnosis”) OR (“Occupational Risks”) OR (“Occupational Exposure”) OR (“Occupational stress”) OR (“Occupational diseases”) OR (“Risk for occupational illness”))
Cochrane Library	((“Nursing professionals”) OR (“Nursing Team”)) AND ((“Causality”) OR (“Etiology”)) AND ((“Nursing diagnosis”) OR (“Occupational Risks”) OR (“Occupational Exposure”) OR (“Occupational stress”) OR (“Occupational diseases”) OR (“Risk for occupational illness”))
EMBASE	((“Nursing professionals”) OR (“Nursing Team”)) AND ((“Causality”) OR (“Etiology”)) AND ((“Nursing diagnosis”) OR (“Occupational Risks”) OR (“Occupational Exposure”) OR (“Occupational stress”) OR (“Occupational diseases”) OR (“Risk for occupational illness”))
Latin American and Caribbean Literature in Health Sciences (LILACS)	((“Nursing professionals”) OR (“Nursing Team”)) AND ((“Causality”) OR (“Etiology”)) AND ((“Nursing diagnosis”) OR (“Occupational Risks”) OR (“Occupational Exposure”) OR (“Occupational stress”) OR (“Occupational diseases”) OR (“Risk for occupational illness”))
Spanish National Institute for Occupational Health and Safety (INSST)	Nursing professionals AND Risk for occupational illness
Spanish National Institute for Occupational Health (INST)	Nursing professionals AND Risk for occupational illness

### Eligibility criteria

The following inclusion criteria will be adopted: studies addressing workers aged at least 18 years old, up to retirement age, and presenting etiological factors consistent with the susceptibility defined by the Nursing diagnosis researched. Additionally, the studies must include analyses related to identifying, defining and associating factors with the *Risk for Occupational Illness*. Exclusion criteria: studies exclusively presenting etiological factors unrelated to Nursing professionals or not addressing the research question. Duplicate studies will be counted only once.

### Data management

In order to ensure methodological rigor and minimize selection bias, study selection will be conducted independently and under active blinding by two reviewers using Rayyan – Intelligent Systematic Review software (https://rayyan.ai/). During the screening process, all studies will be evaluated based on their titles and abstracts, with inclusion determined at this initial stage. Disagreements between the reviewers will be solved through consultation with a third reviewer.

The methodological quality of the studies selected will be appraised using the JBI critical appraisal checklists specific to each study design, as recommended in the JBI Manual for Evidence Synthesis [16]. In the absence of a standardized JBI cutoff score, the inclusion decisions will follow a reviewer-established threshold of ≥6 points. The scores will be calculated as the arithmetic mean based on the number of checklist items applicable to each study type.

### Risk of bias assessment corresponding to the studies

Given that the systematic review will include studies with different designs, their risk of bias will be assessed using the most appropriate tools for each type of study. Non-randomized cohort and case-control studies will be evaluated using ROBINS-I, cross-sectional ones will be assessed using the Joanna Briggs Institute Critical Appraisal Checklist, and randomized controlled trials will be evaluated using the Cochrane RoB 2 tool. All assessments will be conducted independently by two reviewers, with disagreements solved through consultation with a third reviewer.

### Data extraction

Data extraction will be performed using a structured tool developed in Microsoft Excel 2019. This tool will record variables related to study identification, methodological characteristics, elements pertinent to the causal factor analysis of *Risk for Occupational Illness*, antecedents and defining attributes, main findings and key conclusions from each study. Furthermore, it will identify etiological factors and at-risk populations reported in the literature that are not currently included in NANDA-I (Table 3).

**Table 3:** Structured data extraction instrument.

Data extraction instrument						
***Identification of studies:***						
Title	Data source	Country	Language	Year	Authors	Area
***Methodological aspects:***						
Study design		Type of approach		Study objective		Level of evidence
***Elements of the analysis:***						
Causal factors (Which are the etiological factors associated with the *Risk for Occupational Illness* in Nursing professionals?)						
Antecedents of the *Risk for Occupational Illness*:						
Conditions associated with the *Risk for Occupational Illness*:						
Attributes of the *Risk for Occupational Illness*:						
**Main results:**						
**Main conclusions:**						
**Etiological factors evidenced in the study that are not present in NANDA-I:**						
**At-risk populations evidenced in the study that are not present in NANDA-I:**						

### Synthesis of the results

The synthesis of the results will be conducted through iterative readings of the studies included. The findings will be organized into categories developed by the researchers based on conceptual similarities, study design and type of outcome measured. In order to address variability across the studies (including differences in population characteristics, study designs and outcome measurements), the findings will be compared and contrasted across these categories, highlighting patterns, similarities and differences.

The data will be presented descriptively and summarized using tables, charts and other graphical representations, complemented by a narrative synthesis. No quantitative synthesis (meta-analysis) will be performed due to anticipated heterogeneity across study designs and outcomes.

### Analysis of the evidence

The studies included will be appraised according to their level of evidence, following the classification framework established by the JBI. The evaluation will consider the following hierarchy: Level 1 – Experimental studies; Level 2 – Quasi-experimental studies; Level 3 – Analytical observational studies; Level 4 – Descriptive observational studies; and Level 5 – Experts’ opinions and bench research [16]. This appraisal will ease a critical and balanced discussion of the findings, accounting for the evidentiary strength of each study.

### Data collection

Data collection will commence following acceptance of this protocol and will be completed after its publication. The synthesis of the evidence identified in the literature will reflect the most up-to-date information available, encompassing retrospective publications since 2025 — corresponding to the protocol submission year — with no restriction on the retroactive end date.

### Ethics statement

Considering the theoretical nature of this research and the absence of human subjects, submission to a Research Ethics Committee (REC) is waived, as the scientific evidence used is publicly available.

This protocol complies with international ethical standards for systematic and scoping reviews, including responsible use, management and reporting of secondary data.

## Results expected

This protocol is expected to guide the development of an etiology and risk review, aiming to identify the etiological factors — including associated conditions, at-risk populations and risk factors — related to the “*Risk for Occupational Illness*” Nursing diagnosis among Nursing professionals. The results will include a characterization of the studies comprising the sample, with emphasis on the etiological factors associated with this Nursing diagnosis. All research data to be generated from the study based on this protocol, and supporting its conclusions, will be made publicly available in the Mendeley Data repository.

Furthermore, the findings of this review will provide a foundational evidence base to support subsequent stages of the validation process for this Nursing diagnosis within the NANDA-I framework, including concept and content validation, by informing experts’ consensus and highlighting key risk factors and associated conditions.

## Discussion

The motivation for developing this review is based on the need to address stages 3, 4, 7 and 8 of the concept analysis process corresponding to the *Risk for Occupational Illness* Nursing diagnosis. These stages include the following: Identifying possible uses of the concept; Determining critical defining attributes; Identifying antecedents and consequences; and Defining empirical referents [17]. This process seeks to clarify the theoretical underpinnings of the diagnosis, with the potential to revise and enhance its formulation within the NANDA-I taxonomy by recognizing risks that compromise workers’ safety and well-being, thus expanding the focus beyond patients and caregivers to also include Nursing professionals.

The Nursing profession has undergone significant development over time, continuously expanding its practice scope and establishing itself as a cornerstone of health sciences. The increasing number of Nursing professionals underscores the importance of standardized language across care teams, a goal that is eased by using taxonomies such as NANDA-I.

However, for taxonomic systems to effectively support evidence-based practice, ongoing research into occupational illness is essential. Such research contributes to advancing Nursing knowledge and supports the theoretical and empirical validation of this diagnosis within NANDA-I, ultimately improving its level of evidence [10].

The validation process for Nursing diagnoses consists of three sequential phases: Concept analysis, Content validation by expert reviewers, and Clinical validation. The first phase involves identifying essential attributes, antecedents and consequences of the concept. In the second phase, expert reviewers evaluate the content in terms of relevance, consistency, clarity and applicability. Finally, a clinical validation is conducted in real-world care settings [12].

Clearly defining and understanding diagnostic indicators enhances Nursing care breadth and quality by easing a timely identification of human responses and the implementation of effective and coherent Nursing interventions [10, 12, 18].

The findings of this review will be disseminated through peer-reviewed journals in health sciences and are expected to contribute to strengthening the evidence base for the *Risk for Occupational Illness* Nursing diagnosis. This will support its continued inclusion in future editions of the NANDA International classification, ensuring that the diagnosis remains updated and aligned with the most current scientific evidence.

In addition to supporting the concept validation of this Nursing diagnosis, the findings are expected to have real-world implications for the Nursing practice and workplace safety. By identifying key etiological factors and risk conditions, the results can guide the development of targeted preventive strategies, inform institutional policies, enhance risk management protocols and support education and training of Nursing professionals. Ultimately, these outcomes may promote timely recognition and mitigation of occupational hazards, contributing to nurses’ safety, well-being and professional sustainability in diverse healthcare settings.

## Conclusion

This protocol will support an etiology and risk review aimed at synthesizing the current body of evidence and at identifying existing knowledge gaps. By mapping the risk factors associated with occupational diseases, it will be possible to propose targeted interventions tailored to the specific vulnerabilities faced by healthcare professionals. Given the largely preventable nature of many occupational ailments, all factors contributing to occupational susceptibility will be systematically examined and reported.

## Strengths and limitations of this study

- This study will comprehensively review the scientific literature on the etiological factors associated with the Risk for Occupational Illness Nursing diagnosis in Nursing professionals, thereby contributing to a higher level of evidence for this diagnosis;
- The review process will be conducted rigorously, following the criteria established in the JBI Manual for Evidence Synthesis;
- To minimize selection bias, data collection will be performed independently by the researchers using active blinding and supported by software tools for data extraction and selection of studies.

## Supporting information

S1 File Appendix A – Etiology and Risk Review Research Protocol. (PDF)

S2 File PRISMA-P (Preferred Reporting Items for Systematic review and Meta-Analysis Protocols) 2015 checklist: recommended items to address in a systematic review protocol*. (PDF)

## References

[1] Brasil. Lei nº 8.080, de 19 de setembro de 1990. Dispõe sobre as condições para a promoção, proteção e recuperação da saúde, a organização e o funcionamento dos serviços correspondentes e dá outras providências. Brasília (DF): Presidência da República. 1990. https://www.planalto.gov.br/ccivil_03/leis/l8080.htm

[2] Alves NS, Oliveira BA, Carvalho TA, Sampaio LS, Almeida RO. Riscos ocupacionais e seus agravos aos profissionais de enfermagem: revisão integrativa da literatura. Rev Casos Consultoria. 2021;12(1):e25687.

[3] Bermudes WL. Proposta de uma matriz de risco para programa de gerenciamento de risco em atividades rotineiras. Revista Vértices. 2021;23(2):580–9. doi: 10.19180/1809-2667.v23n22021p580-589

[4] Hartikainen E, Solovieva S, Viikari-Juntura E, Leinonen T. Associations of employment sector and occupational exposures with full and part-time sickness absence: random and fixed effects analyses on panel data. Scand J Work Environ Health. 2022;48(2):148–57. doi: 10.5271/sjweh.4003 34850957 PMC9045233

[5] Sun W, Wang H, Li Y. Prevalence of work-related musculoskeletal disorders among nurses: a systematic review and meta-analysis. Int J Environ Res Public Health. 2023;20(4):652. doi: 10.3390/ijerph20040652

[6] Hartikainen E, Solovieva S, Viikari-Juntura E, Leinonen T. Associations of employment sector and occupational exposures with full and part-time sickness absence: random and fixed effects analyses on panel data. Scand J Work Environ Health. 2022;48(2):148–57. doi: 10.5271/sjweh.4003 34850957 PMC9045233

[7] Brasil, Ministério da Saúde. 17º boletim epidemiológico – Acidentes de trabalho com exposição a material biológico em profissionais de enfermagem, 2018–2022. Brasília (DF): Secretaria de Vigilância em Saúde e Ambiente. 2023. https://www.gov.br/saude/pt-br/centrais-de-conteudo/publicacoes/boletins/epidemiologicos/edicoes/2023/boletim-epidemiologico-volume-54-no-17

[8] Aldenora dos Santos J, Paula de Araújo Machado A, Naildo Cardoso Leitão F, Lucas de Souza Ramos J, Elena Guerrero Daboin B, Cilene de Oliveira Cosson I, et al. Nursing assistance systematization: understanding the care implementation process. jhgd. 2023;33(2):231–40. doi: 10.36311/jhgd.v33.14756

[9] Conselho Federal de Enfermagem B. Resolução nº 736, de 17 de janeiro de 2024. Dispõe sobre a implementação do Processo de Enfermagem Internet. Brasília (DF): COFEN. 2024. http://www.cofen.gov.br/resolucao-cofen-no-736-de-17-de-janeiro-de-2024

[10] Herdman TH, Kamitsuru S, Lopes CT. Diagnósticos de enfermagem da NANDA-I: definições e classificação 2024–2026. 13 ed. New York: Thieme Medical Publishers. 2024.

[11] Costa Bezerra D. Systematization of Nursing Assistance: Perspectives of the Applicability in the Care of the Person with Diabetes and Hypertension. AJNS. 2019;8(4):180. doi: 10.11648/j.ajns.20190804.19

[12] Chen L, Gong Y, Yuan L. Health behaviour and its determinants in elderly patients with chronic diseases: evidence from Jiangsu Province, China. BMC Geriatr. 2022;22(1):297. doi: 10.1186/s12877-022-03010-w 35392819 PMC8988547

[13] Moola S, Munn Z, Tufanaru C, Aromataris E, Sears K, Sfetcu R. Systematic reviews of etiology and risk. JBI manual for evidence synthesis. Adelaide: JBI. 2020.

[14] Khan KS, Kunz R, Kleijnen J, Antes G. Five steps to conducting a systematic review. J R Soc Med. 2003;96(3):118–21. doi: 10.1177/014107680309600304 12612111 PMC539417

[15] Cavalcante YA. Validação de conteúdo do diagnóstico de enfermagem “Síndrome Pós-Trauma” no contexto da violência contra a mulher. Fortaleza (CE): Universidade Federal do Ceará. 2022.

[16] Joanna Briggs Institute. The JBI approach: levels of evidence. Adelaide: JBI. 2013. https://jbi.global/sites/default/files/2019-05/JBI-Levels-of-evidence_2014_0.pdf

[17] Walker LO, Avant KC. Strategies for theory construction in nursing. 5th ed. Boston: Pearson Education; 2019.

[18] Sundborg EM, Saleh-Stattin N, Wändell P, Törnkvist L. Nurses’ preparedness to care for women exposed to Intimate Partner Violence: a quantitative study in primary health care. BMC Nurs. 2012;11:1. doi: 10.1186/1472-6955-11-1 22233776 PMC3293728

