# Supplementary material for: Etiological factors of the risk for occupational illness in nursing professionals: An etiology and risk review protocol
Source: PLoS One. 2026 Jan 7;21(1):e0335976. doi: 10.1371/journal.pone.0335976 (PMC12779277; doi:10.1371/journal.pone.0335976)
Supplement: S1 File — (PDF) [file pone.0335976.s001.pdf]

Appendix

Appendix A - Etiology and Risk Review Research Protocol.

<table>
<tr><th colspan="2">REVIEW OF ETIOLOGY AND RISK – STUDY PROTOCOL</th></tr>
<tr><td colspan="2">Theme: Concept analysis of the Nursing Diagnostic term Risk for Occupational Illness in nursing professionals.</td></tr>
<tr><td colspan="2">Objectives: To identify the etiological factors associated with Nursing Diagnosis Risk for Occupational Illness in nursing professionals.</td></tr>
<tr><td colspan="2">Research Question: What are the etiological factors associated with Nursing Diagnosis Risk for Occupational Illness in nursing professionals?</td></tr>
<tr><th colspan="2">IDENTIFICATION OF STUDIES</th></tr>
<tr><td colspan="2">Search Strategies (Data Sources):
● Data source 1: Scientific Eletronic Library Online (SciELO);
● Data source 2: Web of Science;
● Data source 3: ScienceDirect;
● Data source 4: CINAHL (EBSCO);
● Data source 5: MEDLINE/PubMed (via National Library of Medicine);
● Data source 6: Cochrane Library;
● Data source 7: Scopus (Elsevier);
● Data source 8: EMBASE;
● Data source 9: Latin American and Caribbean Literature in Health Sciences;
● Data source 10: National Institute of Security and Health at Work;
● Data source 11: National Institute of Occupational Health.</td></tr>
<tr><td colspan="2">Keywords or indexed descriptors to be used in database searches:
P (population): “Nursing professionals” “Nursing Team”
E (etiology): “Causality”“Etiology”
O (outcome): “Nursing diagnosis” “Occupational risks” “Occupational exposure” “Occupational stress” “Occupational diseases” “Risk for occupational illness”</td></tr>
<tr><th colspan="2">CROSS-REFERENCE IN DATA SOURCES</th></tr>
<tr><td>Scientific Eletronic Library Online (Scielo)</td><td>(*"nursing professionals") AND ("Occupational Exposure") OR ("Occupational stress") OR ("Occupational diseases") OR ("Risk for occupational illness")</td></tr>
</table>

Web of Science	TS=(“Nursing professionals”) OR TS=(“Nursing Team”) AND TS=(“Causality”) OR TS=(“Etiology”) AND TS=(“Nursing diagnosis”) OR TS=(“Occupational Exposure”) OR TS=(“Occupational stress”) OR TS=(“Occupational diseases”) OR TS=(“Risk for occupational illness”)
ScienceDirect	(“Nursing professionals”) AND (“Etiology”) AND ((“Nursing diagnosis”) OR ("Occupational Exposure") OR ("Occupational stress") OR ("Occupational diseases") OR ("Risk for occupational illness"))
CINAHL (EBSCO)	TX(“Nursing professionals”) AND TX(“Etiology”) AND TX(“Occupational Risks”) OR TX(“Risk for occupational illness")
MEDLINE/PubMed	((“Nursing professionals”) OR (“Nursing Team”)) AND ((“Causality”) OR (“Etiology”)) AND ((“Nursing diagnosis”) OR (“Occupational Risks”) OR ("Occupational Exposure") OR ("Occupational stress") OR ("Occupational diseases") OR (“Risk for occupational illness”))
Scopus (Elsevier)	TITLE-ABS-KEY (("Nursing professionals") OR ("Nursing Team")) AND (("Causality") OR ("Etiology")) AND (("Nursing diagnosis") OR ("Occupational Risks") OR ("Occupational Exposure") OR ("Occupational stress") OR ("Occupational diseases") OR ("Risk for occupational illness"))
Cochrane Library	“Nursing professionals” AND Causality OR Etiology AND "Nursing diagnosis" OR "Occupational Risks" OR "Occupational diseases" OR "Risk for occupational illness"
Embase	((“Nursing professionals”) OR (“Nursing Team”)) AND ((“Causality”) OR (“Etiology”)) AND (("Nursing diagnosis") OR ("Occupational Risks") OR ("Occupational Exposure") OR ("Occupational stress") OR ("Occupational diseases") OR ("Risk for occupational illness"))
Latin American and Caribbean Literature in Health Sciences	((“Nursing professionals”) OR (“Nursing Team”)) AND ((“Causality”) OR (“Etiology”)) AND (("Nursing diagnosis") OR ("Occupational Risks") OR ("Occupational Exposure") OR ("Occupational stress") OR ("Occupational diseases") OR ("Risk for occupational illness"))
National Institute of Security and Health at Work	Nursing professionals AND Risk for occupational illness

National Institute of Occupational Health	Nursing Professionals AND Risk for occupational illness

SELECTION OF STUDIES

Inclusion criteria:

- Studies involving workers aged 18 years or older, up to retirement age;
- Studies that identify etiological factors (associated conditions, at-risk populations, and risk factors) related to susceptibility to occupational illness;
- Studies that include analyses focused on the identification, definition, and association of factors with the Nursing Diagnosis *Risk for Occupational Illness*.

Exclusion criteria:

- Editorials, letters to the editor and abstracts;
- Studies that exclusively report clinical indicators related to the nursing diagnosis *Risk for Occupational Illness*, without addressing etiological factors.

Selection strategy:

- To ensure methodological rigor and minimize selection bias, the study selection process will be conducted independently by the reviewers using a blinded approach, supported by Rayyan – Intelligent Systematic Review software.
- During the screening phase, all studies will be evaluated for eligibility, beginning with a preliminary review of titles and abstracts. In cases of disagreement between reviewers, a third reviewer will be consulted to reach a consensus. Full-text articles will then be assessed to support a more accurate and assertive selection.
- Duplicate studies will be identified and counted only once.

DATA MAPPING AND EXTRACTION

- For the critical appraisal of the studies, the standardized checklists provided in the JBI Manual will be used. The inclusion or exclusion of studies will be based on a predefined cut-off score (≥6).
- Data extraction will be performed using a structured instrument specifically developed for this review. The instrument will include variables related to study characteristics, essential attributes, associated conditions, and at-risk populations relevant to the nursing diagnosis *Risk for Occupational Illness*. Additionally, the tool

will identify which etiological factors and at-risk populations reported in the studies are not currently listed in the NANDA-I classification.
**PRESENTATION OF RESULTS**
● Analysis conducted according to the stages proposed by Lopes, Silva, and Araújo (2022);<br>● Study characterization table;<br>● Use of tables to highlight the essential attributes and etiological factors of the *Risk for Occupational Illness*, along with their conceptual and operational definitions.
